# Perforator-based local flaps for cutaneous facial reconstruction

**DOI:** 10.1186/s40902-024-00435-8

**Published:** 2024-08-01

**Authors:** Khaled Mohamed Abdel Azeem, Sarah Mohamed Abdelghany Abdelaal, Mohamed Fathi Abdel Maguid, Philobater Bahgat Adly Awad, Basma Hussein Abdelaziz Hassan, Wael Mohamed El Shaer, Mostafa Fathy Ibrahim Ahmed

**Affiliations:** 1 Plastic and Reconstructive Surgery Department, Faculty of Medicine, Beni-Suef University, Beni Suef, Egypt; 2 General Surgery Department, Faculty of Medicine, Ain Shams University, Cairo, Egypt

**Keywords:** BCC, Perforator-based flaps, Facial soft tissue defects

## Abstract

**Background:**

Despite the advancement of reconstructive surgical techniques, facial defect reconstruction has been always very challenging, aesthetic satisfaction has often been difficult to achieve due to the unique characteristics and complexity of the facial tissue. There have been various options regarding reconstruction and compensation of soft tissue loss all over the body rather than the face. Regardless of whether skin grafts, local flaps, and free flaps were used in the reconstruction process, each of them has its limitations. Beginning with skin grafts results could not always be satisfactory due to contracture, color, and lack of texture Additionally, local flaps have limitations due to mobility and the availability of overlapping skin and tissue, as well as the bulkiness of the pedicle which may need a second staged surgery and lately the difficulty of the free flaps and being a major surgery.

**Results:**

Patients ages ranged between 23 and 77 years old, with a mean age of 58.33 ± 12.47. As regards the patients’ sex, 63.3% of our patients were males and 36.7% were females. Co-morbidities were found in 60% of cases (DM 23.3%, HTN 20%, HCV 3.3%, cardiac 3.3%).

Most flaps were facial artery perforator flaps 53.3%, then transverse facial artery 26.7%, superficial temporal artery 10%, angular artery 6.7%, and supra-trochlear artery 3.3%.

Twenty-ix cases representing 86.7% of cases went uneventful, while complications showed in 4 cases representing 13.3% of cases, 1 case (3.3%) showed venous congestion that was relieved within 24 h after 2 suture releases, another case (3.3%) showed wound dehiscence that was improved after 2 days with regular dressings, the third patient (3.3%) had recurrence after 4 months that was treated by excision and grafting, while last patient (3.3%) had inadequate excision that was treated by radiotherapy. No bleeding or infection occurred. Also, we observed no correlation between flap length and complications. As regards the functional point of view, all patients showed no functional impairment at the donor site, and only one case showed functional impairment at the recipient site.

As regards patient satisfaction, all 30 patients achieved positive satisfaction scores using the Likert scale, 18 cases were satisfied, and 12 cases were very satisfied.

**Conclusion:**

The use of perforator-based flaps can provide a more effective and aesthetically pleasing solution for the reconstruction of small to moderate facial defects, provided that a reliable Perforator is accurately identified and executed by an experienced surgeon.

## Background

The facial area is a major factor in daily interactions, as it is the expression of emotions, beauty, and self-identity. The self-image and self-esteem of an individual are largely determined by their facial appearance, and any injury affecting these features necessitates special consideration. Facial defects may be the result of injury or surgical excision [1]. In the emergency department, traumatic facial soft tissue injuries account for approximately 10% of all visits. When examining statistics regarding the occurrence of human cancers, it is estimated that millions of people are affected annually [2, 3]. However, skin cancers are the most diagnosed malignancies in humans, with one in every three malignancies being skin cancer; they are often localized on the face [4].

Perforator flaps have opened up a whole new horizon for the plastic surgeon to choose flaps for better function and cosmesis as our understanding of the architecture of blood flow to the skin has improved [5].

The aim of this study is to assess the role of perforator-based flaps for cutaneous facial reconstruction from aesthetic and functional points of view on a scale of 30 patients.

## Methods

A prospective clinical study including a group of 30 patients, whose mean age was 58.33 ± 12.47, were chosen to undergo perforator-based flap surgery at the Plastic and Reconstructive Surgery Department, University Hospital, and other non-governmental hospitals. The patients had small to moderate size facial defects (5–6 cm) resulting from post-tumor resection.

### Operative technique

All the operations were performed in the operating room, 27 cases were performed under general anesthesia and 3 cases under local anesthesia.

- Tumor excision

**Fig. 1:**
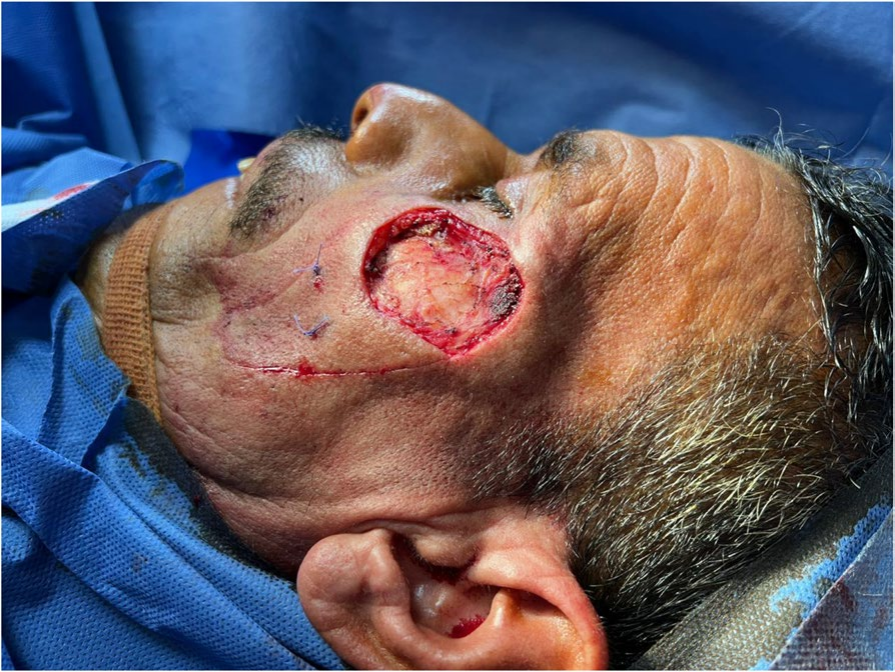
Tumor excision with wide local resection

Existing skin tumors were excised with wide surgical margins (Fig. 1).

- Perforator localization

Eight MHZ handheld doppler was used to identify all the perforators surrounding the defect, and several perforators were marked.

- Perforator selection

Exploration of the chosen perforator was done before raising the flap to make sure that it was suitable for the vascular supply of the flap and for its proposed movement into the defect. Then, the final perforator was selected by the reliability of the caliber and length among the identified perforators.

- Flap dissection

**Fig. 2:**
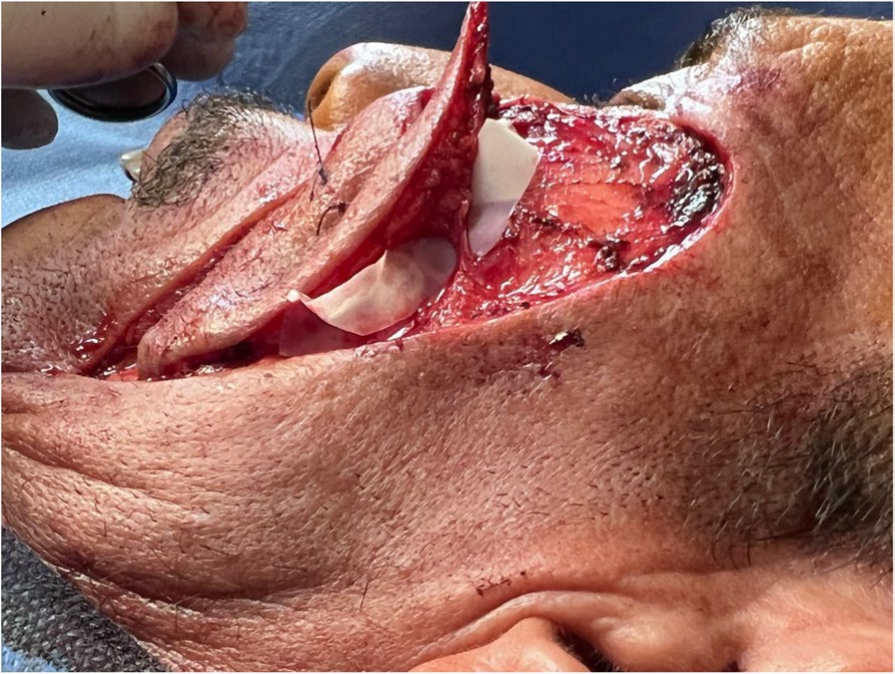
Perforator skeletonization

A flap was designed adjacent to a defect based on the amount of tissue that remained for reconstruction. The skin paddle was designed slightly larger than the defect size to enable insetting with minimal tension. The flaps were elevated and dissected meticulously using loupe magnification × 3.5 and inset into the defect areas along the axis of the perforator by rotation, transposition, or advancement. If a flap needed rotation for insetting, the perforator artery was dissected more meticulously. Whether the perforator would be skeletonized (Fig. 2) or not, would be governed by the needed movement of the flap to be in set without any compromise of its blood supply. If advancement was sufficient, perforator skeletonization was unnecessary.

- Wound closure

**Fig. 3:**
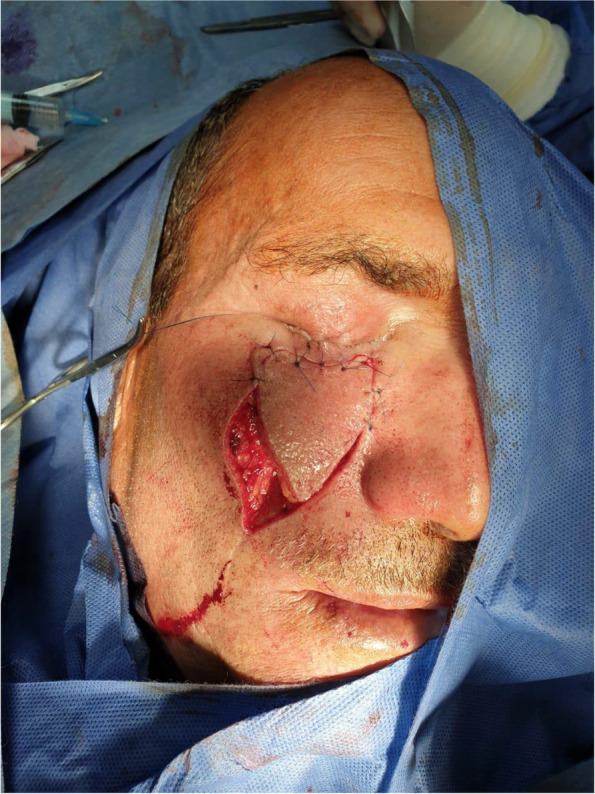
In the setting of the flap

The donor site was closed directly in two layers (Fig. 3), the dermis, and the skin, with minimal undermining. The flap was sutured in two layers in a tension-free manner, after which a slightly compressive dressing was applied. The skin was sutured in two layers with a 5.0 resorbable monofilament suture in the dermis and a monofilament 5.0 prolene in the skin. In the first three patients, the subcutaneous drain was placed underneath the flap for drainage, but we found that this was not necessary and was abandoned in the successive flaps. Paper tape and a light dressing were placed on the scar and suture removal was planned in 7 days at the clinic.

### Post-operative follow-up

A histopathological examination was done for the excised lesions. The patient stayed in the hospital for 48 h and was given antibiotics, antiedematous, analgesics, and proper fluids. Patients were followed up in the outpatient clinic after 1 week for suture removal and then checked in on a monthly, quarterly, and annually thereafter, depending on the pathology involved. Four cases showed complications including congestion, wound dehiscence, inadequate excision, and recurrence.

### Flap assessment

Through clinical assessment as color, warmth, tension on the flap, and blood flow (by capillary filling test).

### Statistical analysis

Data was analyzed using the Statistical Package for Social Sciences (SPSS)version 24. A *p* value < 0.05 was considered significant.

### Methods of evaluation

Aesthetic and function aspects were evaluated by both patients measured by the “Likert scale” instrument and a group of 3 independent consultants utilized a visual analog scale (scores 1–10).

#### Aesthetic aspect (contour, color match, scar)

##### Post-operative subjective satisfaction for surgeons

Utilizing a visual analog scale (scores 1–10) with pre-and post-operative photos during outpatient consultations 3 and 6 months following surgery, independent consultants were asked to rate the post-operative look on a visual analog scale ranging from 1 to 10 as regards contour, color match, and score.

##### Post-operative patient satisfaction

Was measured by the Likert scale instrument, translated into Arabic to evaluate patient satisfaction using visual analog.

#### Functional aspect

##### Independent surgeons

Evaluation was done regarding the motor affection of both donor and recipient and the compatibility of the flap with the vital function of the recipient site.

##### Patients

Were asked during outpatient consultations 3- and 6 months following surgery if they complained of any disability or lack of function in both donor and recipient sites following the operation.

## Results

In our study, the age of the patients ranged between 23 and 77 years old with a mean age of 58.33 ± 12.47. As regards the patients’ sex, 63.3% of our patients were males and 36.7% were females (Table 1). Co-morbidities were found in 60% of cases (DM 23.3%, HTN 20%, HCV 3.3%, cardiac 3.3%) (Table 2).

**Table 1:** Demographic data of the studied patients

		No. = 30
Age	Mean ± SD	58.33 ± 12.47
	Range	23–75
Gender	Female	11 (36.7%)
	Male	19 (63.3%)
Occupation	Household	13 (43.3%)
	Student	1 (3.3%)
	Employee	4 (13.3%)
	Farmer	8 (26.7%)
	Driver	4 (13.3%)
Habits	No special habits	14 (46.7%)
	Smoker	16 (53.3%)

**Table 2:** Co-morbidities of the studied patients

		No	%
Co-morbidities	No	18	60.0%
	Yes	12	40.0%
DM (controlled)	No	23	76.7%
	Yes	7	23.3%
HTN (controlled)	No	24	80.0%
	Yes	6	20.0%
HCV	No	29	96.7%
	Yes	1	3.3%
Cardiac (controlled)	No	29	96.7%
	Yes	1	3.3%

In our work regarding the site, the cheek was the most common site (56.7%), the nose was the second common site (23.3%), then the temple (20%).

we were able to cover a maximum defect surface area of about 18 cm^2^, and most of the defects were between 10 and 15 cm^2^ (Table 3). Twenty-one cases were reconstructed using advancement flap, 15 cases of them were V–Y advancement flap, 6 cases were nasolabial advancement flap, 7 cases were rotational, and 2 cases were propeller.

**Table 3:** Site and size of the defect

			No. = 30
Side of defect		Right	12 (52.2%)
		Left	11 (47.8%)
Site of defect		Cheek	17 (56.7%)
		Nose	7 (23.3%)
		Temple area	6 (20.0%)
Size of defect	Width	Mean ± SD	2.85 ± 0.74
		Range	2–4.5
	Length	Mean ± SD	2.98 ± 0.89
		Range	2–5

As regards anesthesia, 90% of patients had general anesthesia and 10% of them had local anesthesia.

The majority of flaps were facial artery perforator flaps 53.3%, transverse facial artery 26.7%, superficial temporal artery 10%, angular artery 6.7%, supra-trochlear artery 3.3% (Table 4).

**Table 4:** Type of flap, pathology, and source vessel of the studied patients

		No	%
Type of flap	V–Y Nasolabial advancement flap	21	70.0%
	Rotational flap	7	23.3%
	Propeller flap	2	6.7%
Pathology	BCC	30	100.0%
Source vessel	Facial artery	16	53.3%
	Transverse facial artery	8	26.7%
	Angular artery	2	6.7%
	Superficial temporal artery	3	10.0%
	Supra-trochlear artery	1	3.3%

Twenty-six cases representing 86.7% of cases went uneventful, while complications were shown in 4 cases representing 13.3% of cases, 1 case (3.3%) showed venous congestion, another case (3.3%) showed wound dehiscence, a third patient (3.3%)had recurrence after 4 months, while last patient (3.3%) had inadequate excision. No hematoma or infection occurred (Table 5).

**Table 5:** Complications of the studied patients

		No	%
Complications	Non-complicated	26	86.7%
	Complicated	4	13.3%
Complications	Non-complicated	26	86.7%
	Recurrence (surgical excision and graft)	1	3.3%
	Inadequate excision (radiotherapy)	1	3.3%
	Dehiscence (was left for 2 days)	1	3.3%
	Congestion (relieved by 2 suture release)	1	3.3%

As regards evaluation, the flaps were evaluated from 2 aspects:

### 1) The aesthetic aspect (contour, color match, and scar) (Table 6)

**Table 6:** A questionnaire of surgeons’ aesthetic satisfaction score

Category	Condition	Score
Contour	Poor	1
	Fair	2
	Good	3
	Excellent	4
Color match	Distinguishable	1
	Acceptable	2
	Not distinguishable	3
Scar	Markedly visible	1
	Minimally visible	2
	Barely visible	3

#### Post-operative subjective aesthetic satisfaction for surgeons

Utilizing a visual analog scale (scores 1–10) with pre-and post-operative photos during outpatient consultations 6 months following surgery, independent consultants were asked to rate the post-operative look on a visual analog scale ranging from 1 to 10 as regards contour, color match, and score. Ten patients were given a 9 as a score regarding aesthetic aspect by surgeons independent from the study, 8 were given an 8, and 12 were given a score of 7 (Table 7).

**Table 7:** Surgeons’ satisfaction score and patient satisfaction according to the Likert scale of the studied patients

		No. = 30
Surgeons' satisfaction score	Median (IQR)	8 (7–9)
	Range	7–9
Patient's Satisfaction score	Satisfied	18 (60.0%)
	Very satisfied	12 (40.0%)

#### Post-operative patient aesthetic satisfaction

**Fig. 4:**
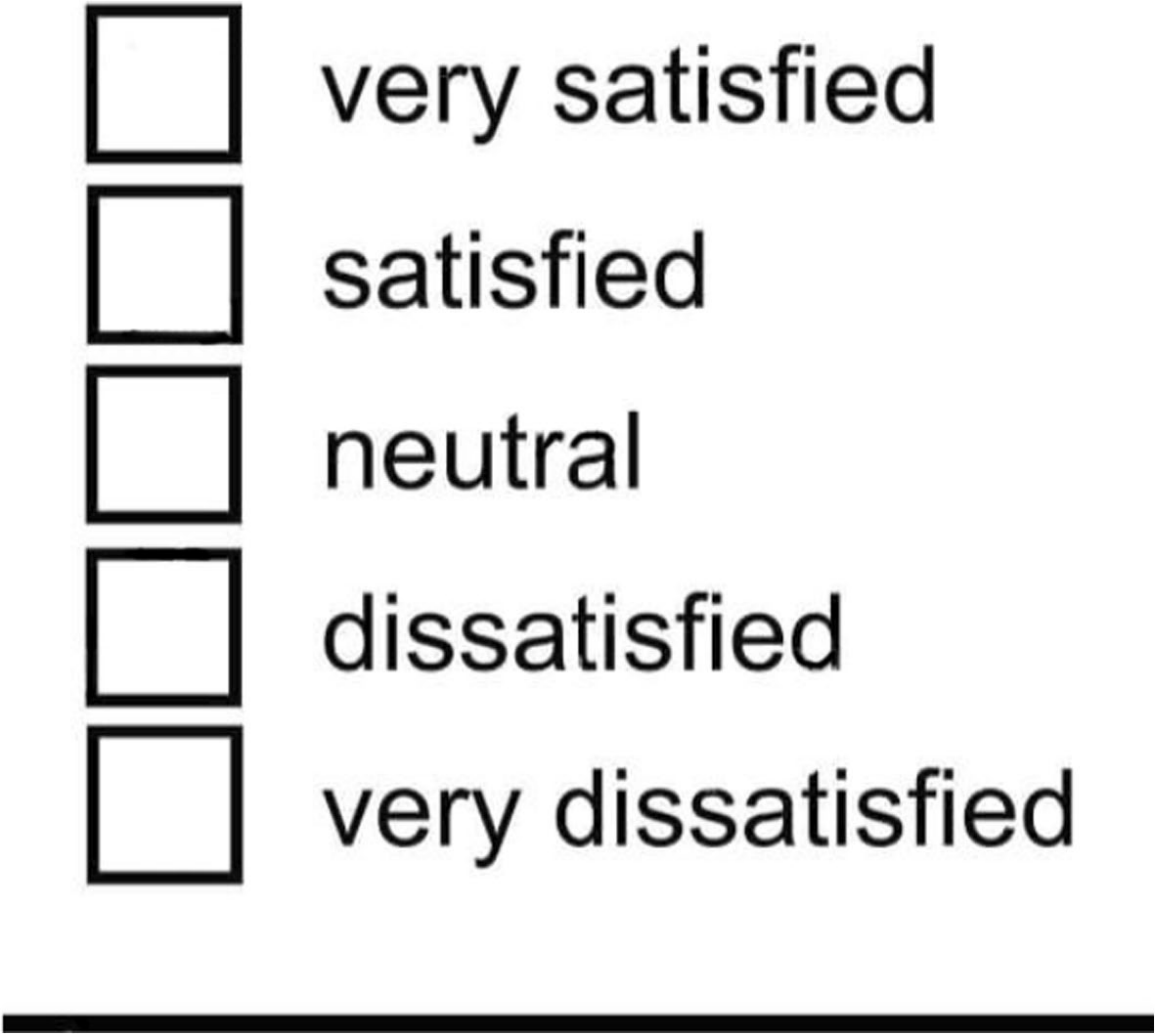
A questionnaire of patients’ aesthetic satisfaction (Likert scale)

Was measured by the Likert scale instrument (Fig. 4), translated into Arabic to evaluate patient satisfaction using visual analogue.

Eighteen patients were satisfied and twelve were very satisfied.

### Functional aspect

#### Independent surgeons

Evaluation was done regarding motor affection of both donor and recipient, the compatibility of the flap with the vital function of the recipient site, i.e., Periorbital reconstruction had obstacles after excision of malignant skin tumors such as achieving symmetry, stable eyelid margin, smooth internal surfaces providing appropriate vertical and horizontal eyelid measurements for maximal function, adequate eyelid closure to avoid exposure sequelae and retaining normal tension.

In our study, two patients were documented to have recipient site morbidity seen as loss of function of ala nasi.

#### Patients

Were asked during outpatient consultations 6 months following surgery if they complained of any disability or lack of function in both donor and recipient sites following the operation and we found that two patients reported decreased nasal opening on the side of surgery.

## Discussion

In our study, the ages of the patients ranged between 23 and 77 years old. This goes well with other studies [6, 7]. in which their age ranges were 48–82 years, and 29–84 years respectively. 63.3% of our patients were males and 36.7% were females (Table 1). In the study of Aksam et al. [6], 69.1% were males and 30.9% were females while Moio et al. [7] operated on 58% male patients and 52% females. This prevalence is consistent with many studies [8–11]. In contrast, others [12, 13] observed that BCC is more common in females. That was supported by a study done by Mancuso et al. [14] that revealed that sex hormones like estrogen may play a role in skin cancer development, but this relationship has yet to be thoroughly investigated. In our work, no co-morbidities were found in 60% of cases (Table 2) in disagreement with Aksam et al. (2017) who reported that all patients had some kind of comorbidity: hypertension at 28.5%, diabetes at 21.4%, and smoking history at 83.3%.

The maximum defect surface area that we were able to cover was about 18 cm^2^, and most of the defects were between 10 and 15 cm^2^, which is considered a great success for this technique Brunetti et al. [15] were able to reconstruct face defect surface area up to 16 cm^2^.In our study, the cheek was the most common site (56.7%), then the nose (23.3%), then the temple (20%). In literature this point is debatable but what is agreed by most [2, 6] that nose and check are the most common sites (Table 3).

In our study the majority of flaps were facial artery perforator flaps 53.3%, then transverse facial artery 26.7%, superficial temporal artery 10%, angular artery 6.7%, supra-trochlear artery 3.3% (Table 4). Our work was supported by Gunnarsson et al. [2] where 80% of cases were facial artery perforator flaps.

Regarding the type of flaps, it was observed that they differ from one place to another and we think that returns to the surgeon’s preferences rather than other parameters. In our work, 21 cases were reconstructed using advancement flap, and 15 cases of them were V–Y advancement flap, 6 cases were nasolabial advancement flap, 7 cases were rotational, and 2 cases were propeller (Table 4), while Rao and Shende et al. [11] done VY advancement flap on (34/70) patients, while the nasolabial flap was used on (24/70) patients, the median forehead flap was used on (8/70) patients, and the regular forehead flap cover was used on (4/70) patients., while in another study [2]. The flaps were designed as a propeller in the majority of cases (76%) and advancement V–Y in the remaining 24%.

**Fig. 5:**
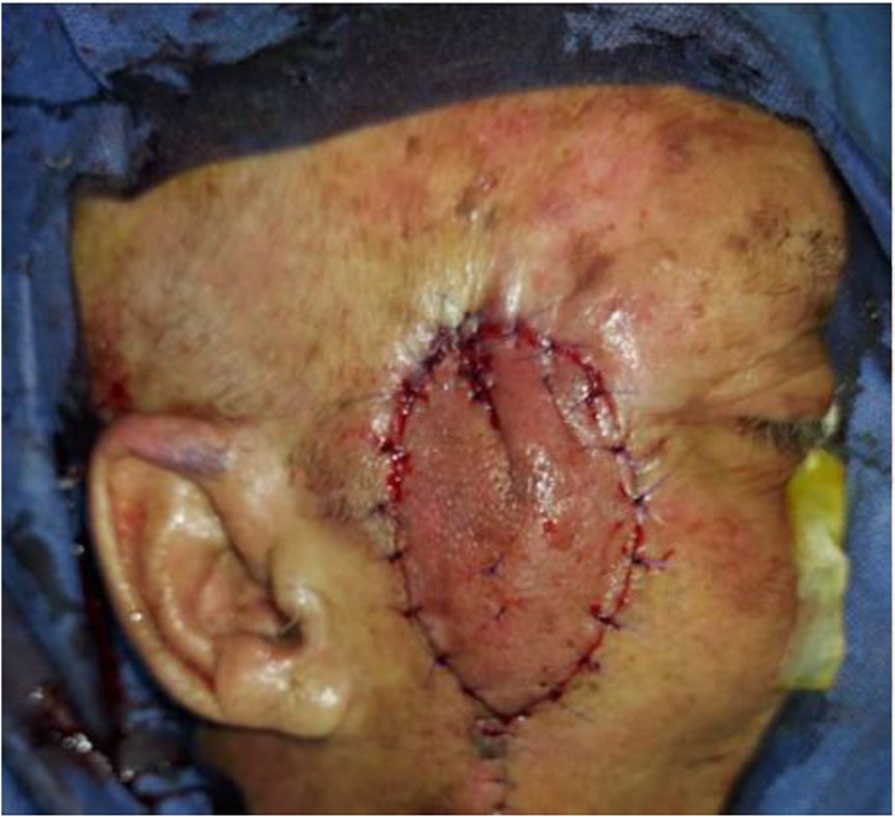
Post-operative flap congestion

**Fig. 6:**
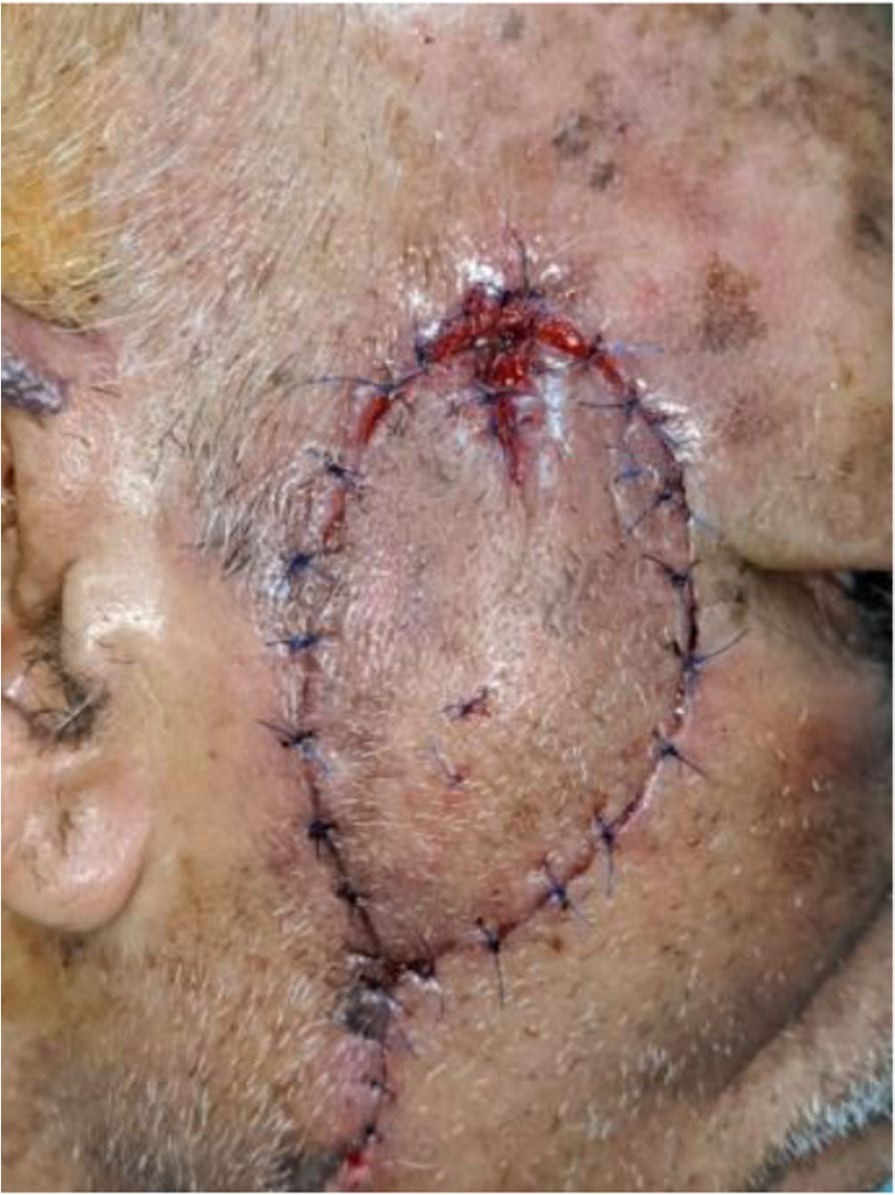
Relief of post-operative congestion by release of 2 sutures

In our study complications rate was 13.3%, 1 case (3.3%) showed venous congestion (Fig. 5) that was relieved within 24 h after 2 suture releases (Fig. 6), another case (3.3%) showed wound dehiscence that was improved after 2 days with regular dressings, the third patient (3.3%)had recurrence after 4 months that was treated by excision and grafting, while last patient (3.3%) had inadequate excision that was treated by radiotherapy (Table 5). No bleeding or infection occurred, in our study, all flaps were fully survived, with no cases of partial or total flap loss observed. The flap healed satisfactorily, with no revision required in the initial post-operative period. There is a lack of literature reporting on the complication rate, however, the majority of studies [2, 3, 16, 17] indicated that the complication rate was within the range of 5.36–39.1%. venous congestion is the most reported complication in Perforator Local Flaps with different management techniques in our study the only patient who had venous congestion(Fig. 5) showed magnificent improvement after suture release (Fig. 6). In Aksam`s study [6], 6 of 42 patients had venous congestion and he used subcutaneous heparin injections and he mentioned that all resolved without the need for any further intervention also in a study [16] conducted on 30 patients 10% of patients had venous congestion which was resolved by medication. Regarding recurrence and inadequate excision, we hypothesize that due to the inability to do a frozen histopathology section intra-operative which we consider one of our study limitations finally, we observed no correlation between flap length and complications in disagreement with Goutam Guha [3] whose clinical study involved 23 patients and showed the flap complication rate 39.1% and he concluded a strong correlation between complications and flap length.

As regards aesthetic and function aspects both were evaluated by both patients and a group of 3 independent consultants, we achieved satisfactory results for both patients and surgeons, in agreement with other studies [2, 18] which reported single-stage reconstruction with perforator-based flap as a highly satisfactory procedure with high aesthetic outcome and patient satisfaction (Table 7).

## Conclusion

The use of perforator-based flaps can provide a more effective and aesthetically pleasing solution for the reconstruction of small to moderate facial defects, provided that a reliable Perforator is accurately identified and executed by an experienced surgeon.

**Fig. 7:**
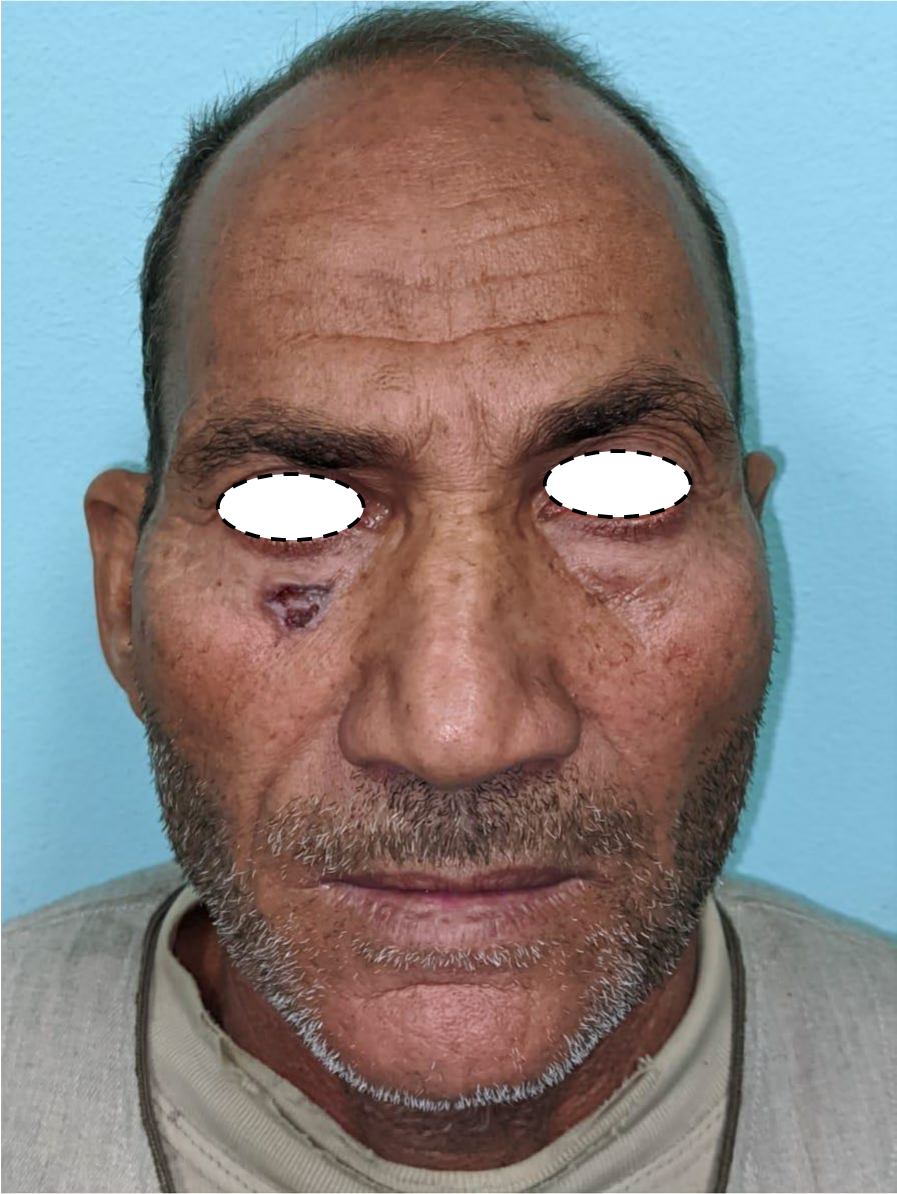
Patient (1): anteroposterior view prior to excision

**Fig. 8:**
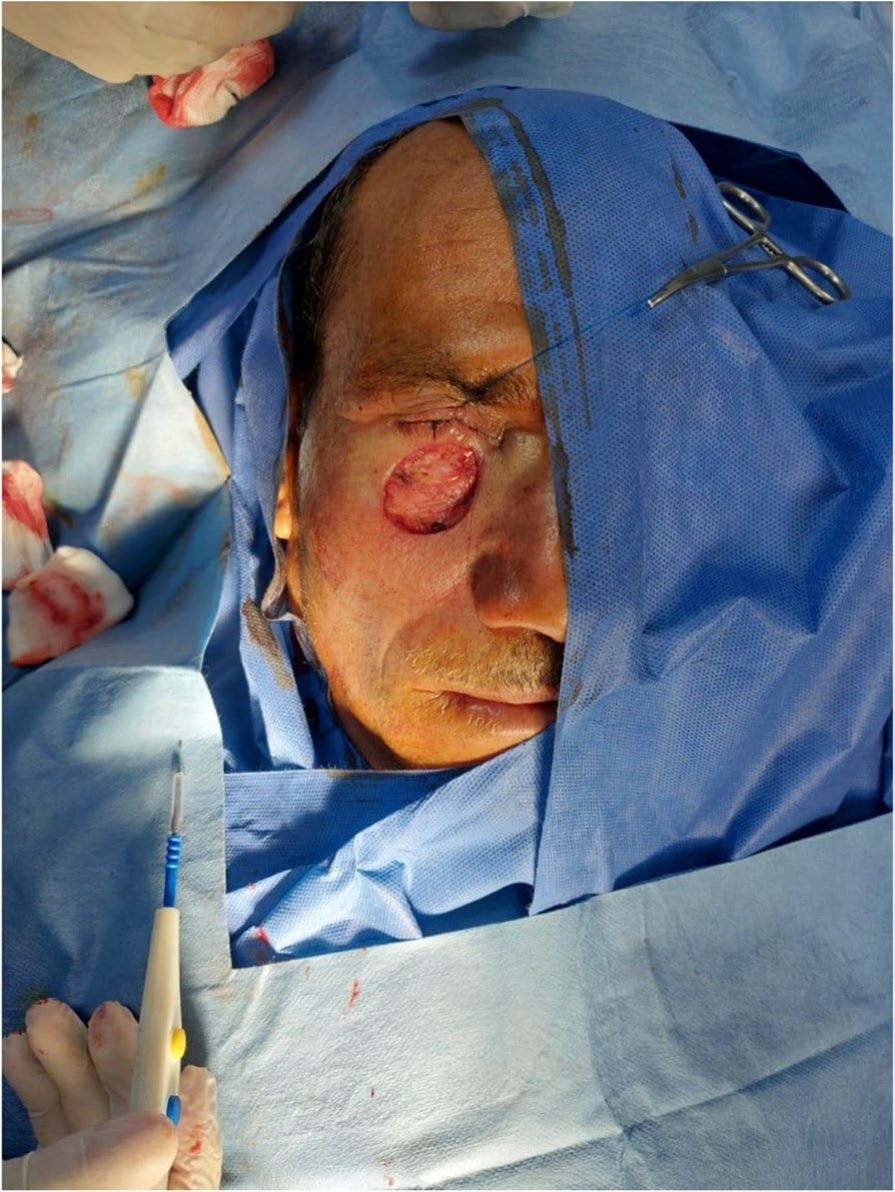
Patient (1): the size of the defect after excision with safety margin

**Fig. 9:**
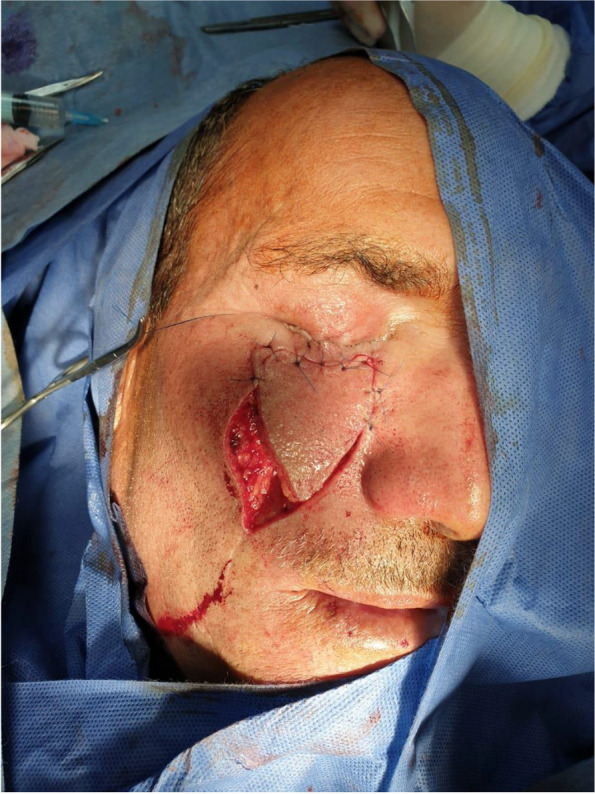
Patient (1): in setting of the flap

**Fig. 10:**
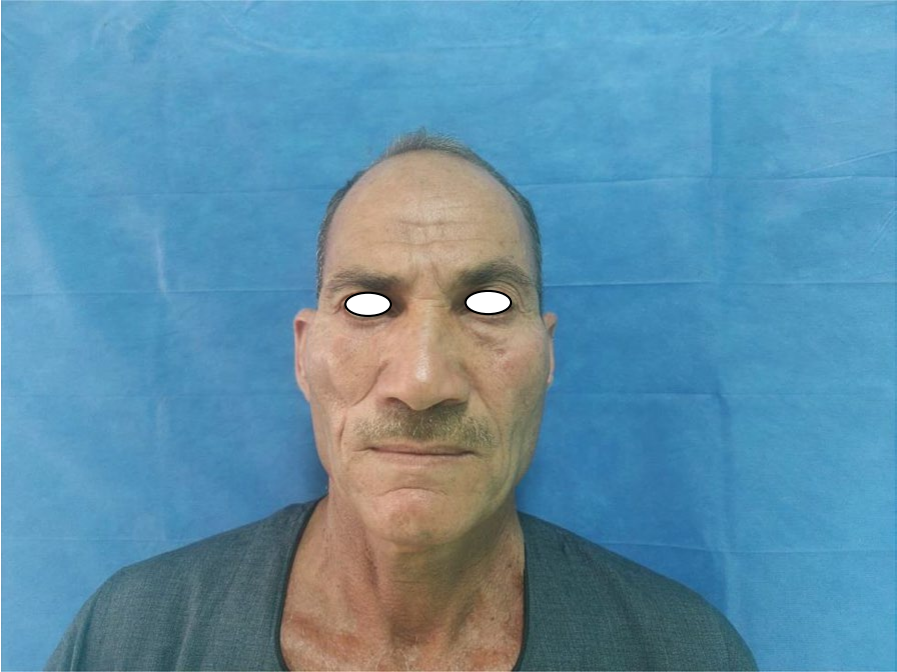
Patient (1): after healing of the flap in post-operative follow-up

Patient 1: a 62-year-old male patient, a smoker, medically free, had an ulcer in his right cheek 6 months ago (Figs. 7, 8, 9, and 10).

**Fig. 11:**
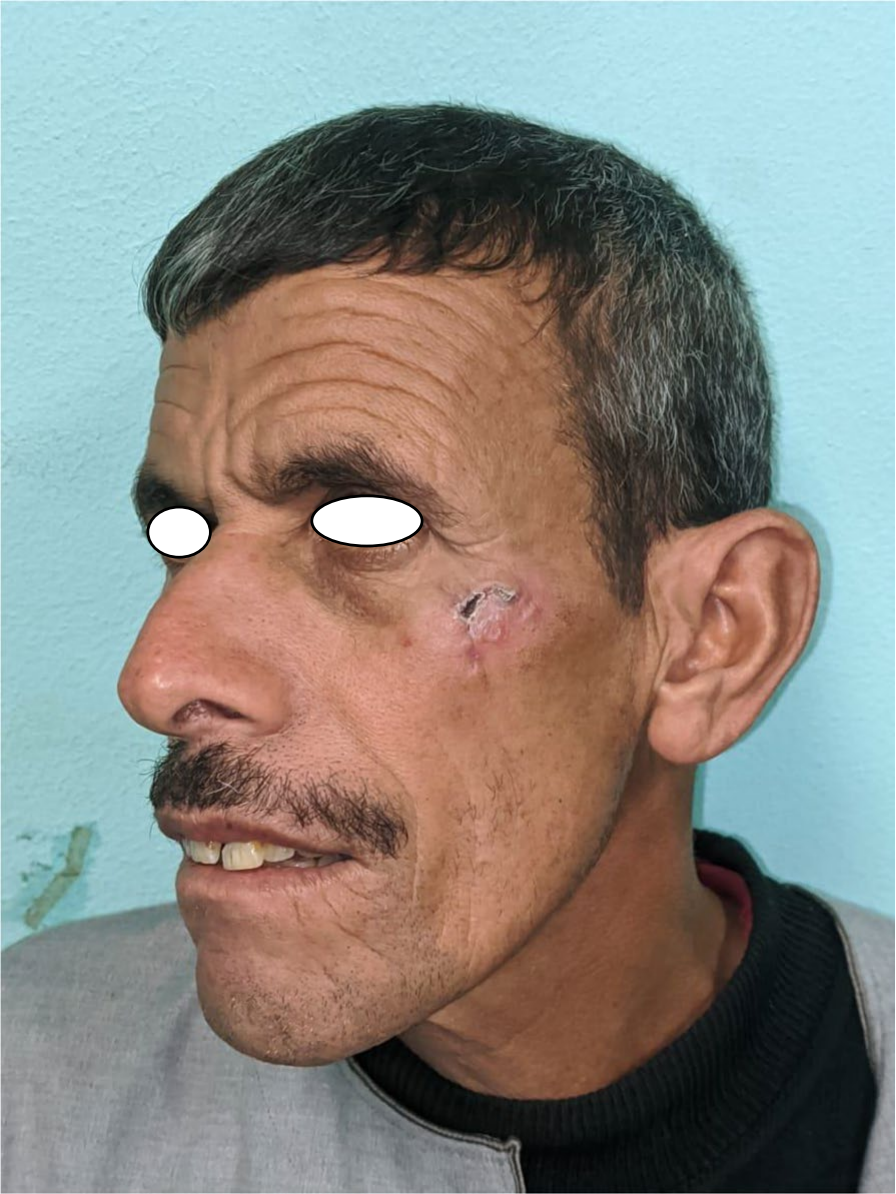
Patient (2): oblique view of the lesion prior to excision

**Fig. 12:**
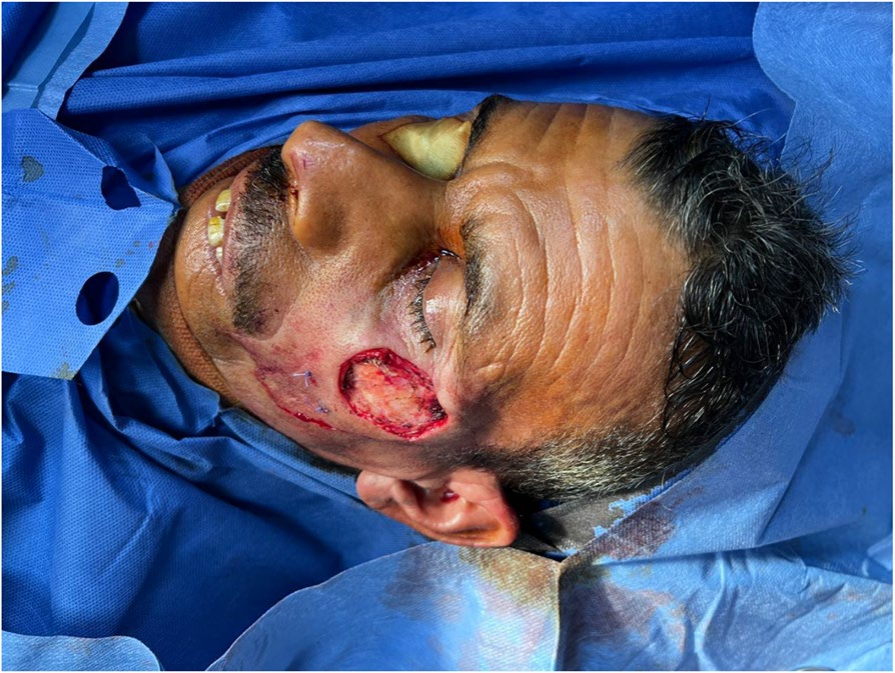
Patient (2): the size of the defect after excision with safety margin

**Fig. 13:**
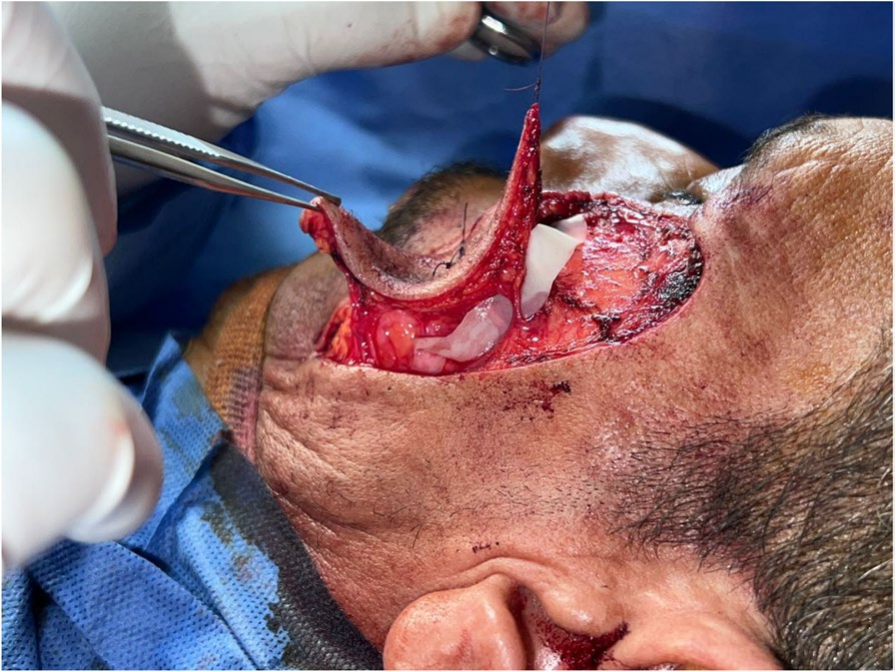
Patient (2): in setting of the flap

**Fig. 14:**
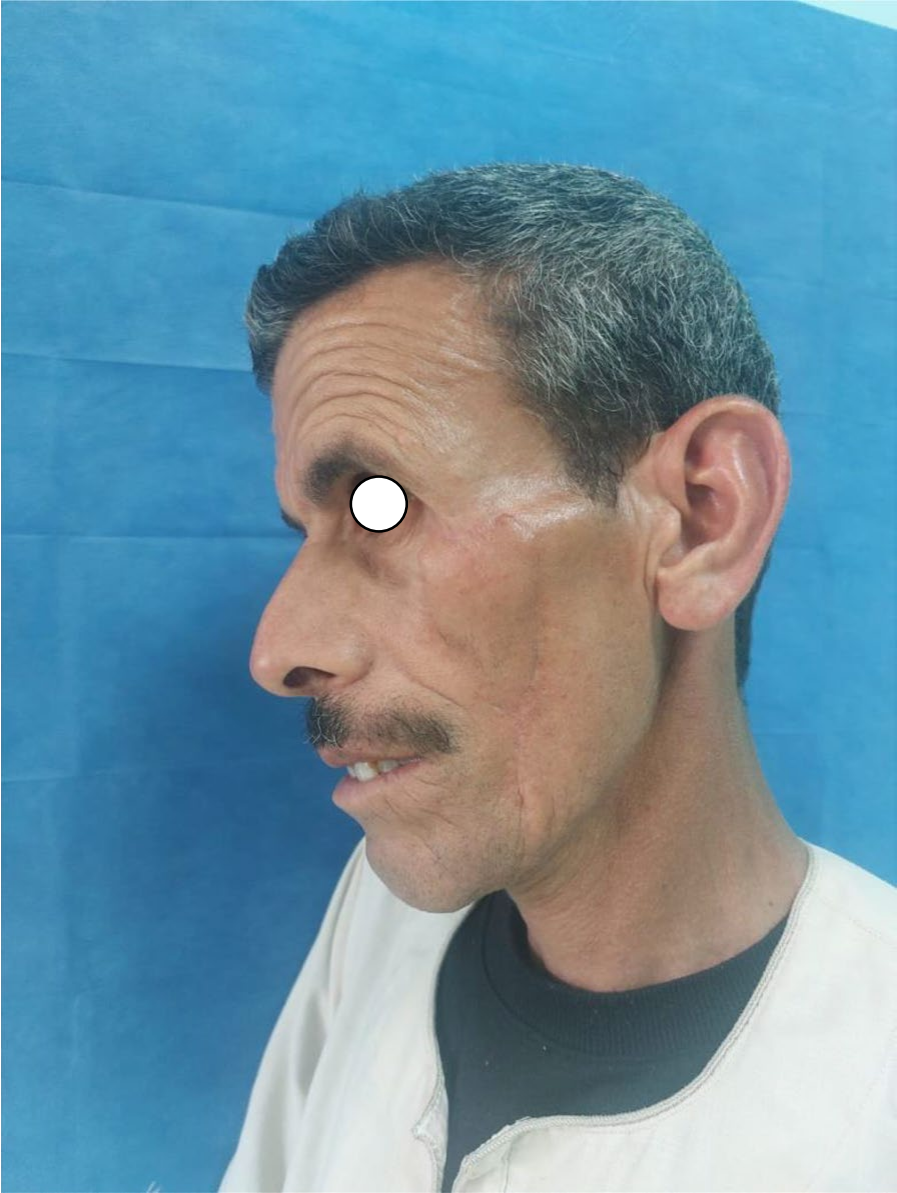
Patient (2): after healing of the flap in post-operative follow-up

Patient 2: a 53-year-old male patient, medically free, ulcer for 4 months on the left cheek (Figs. 11, 12, 13, and 14).

**Fig. 15:**
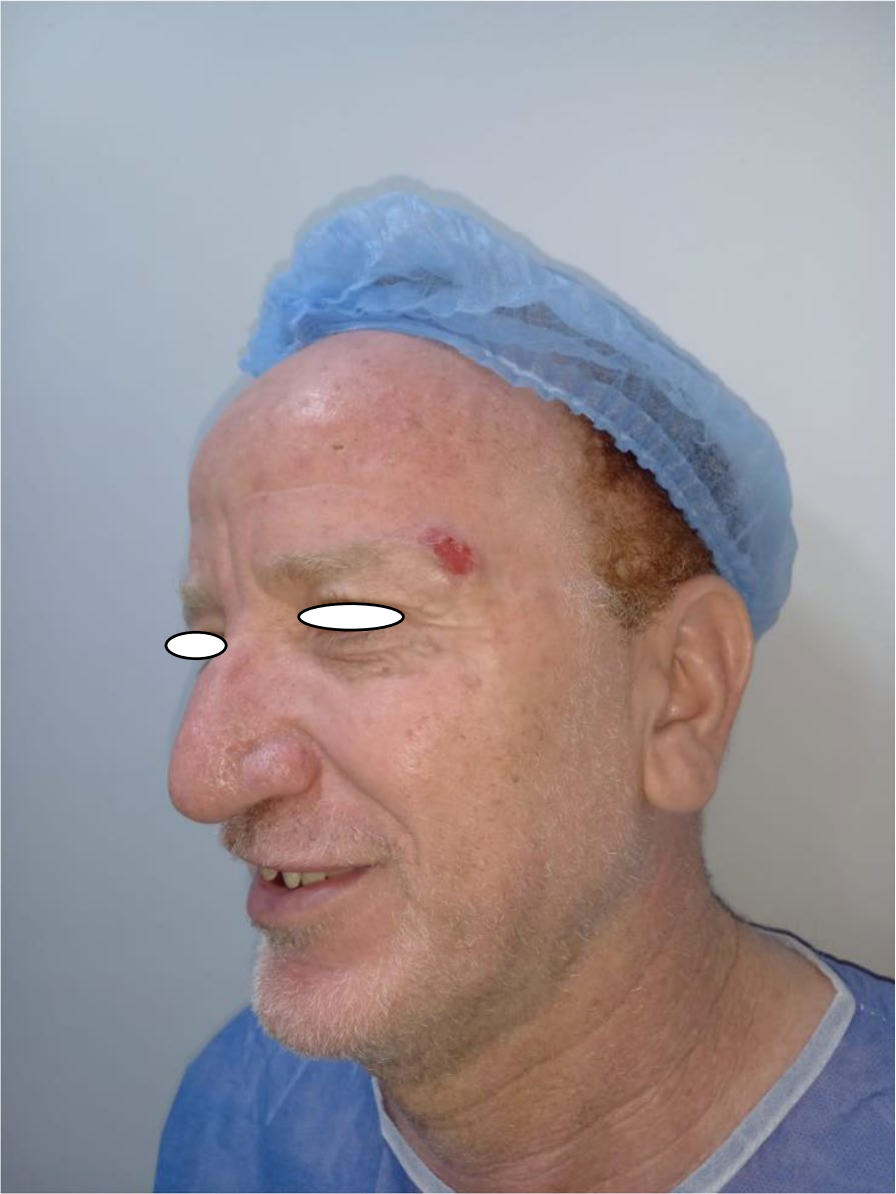
Patient (3): oblique view of the lesion prior to excision

**Fig. 16:**
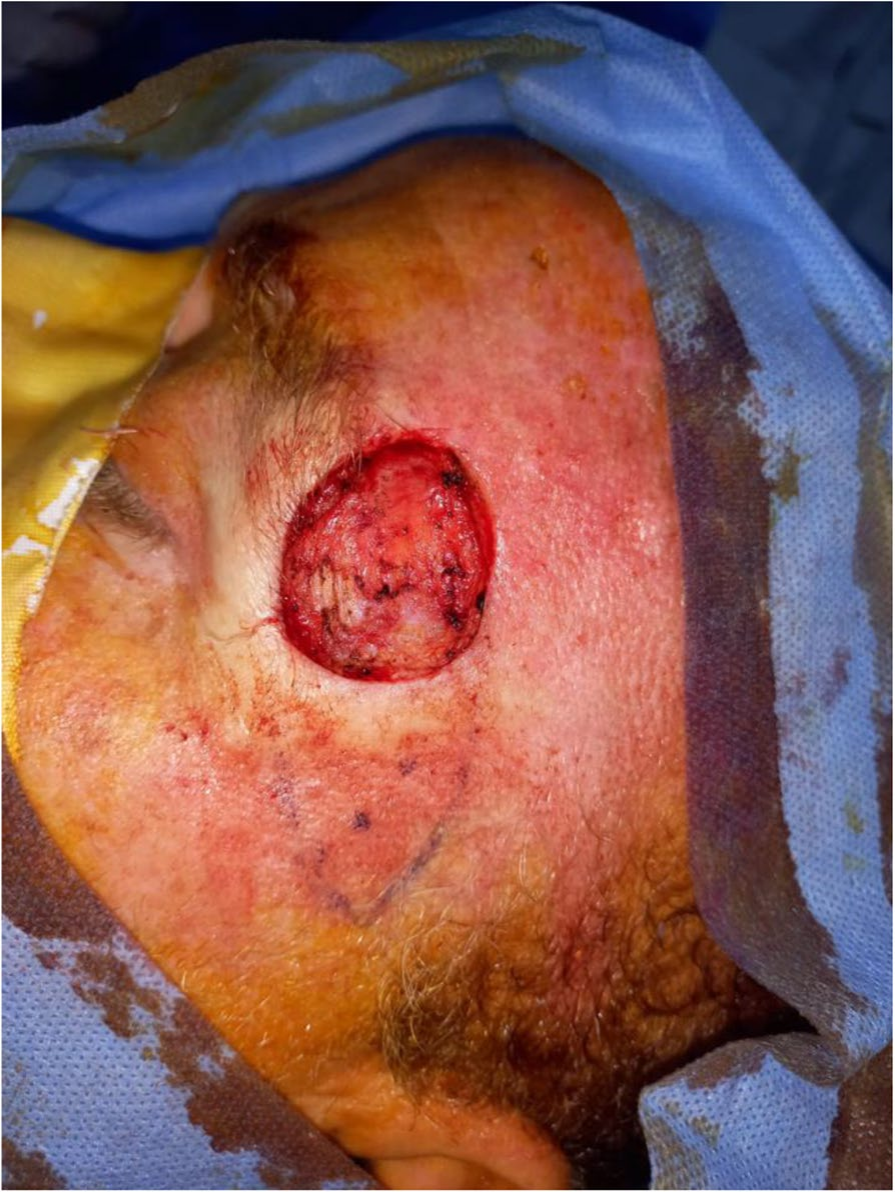
Patient (3): the size of the defect after excision with a safety margin

**Fig. 17:**
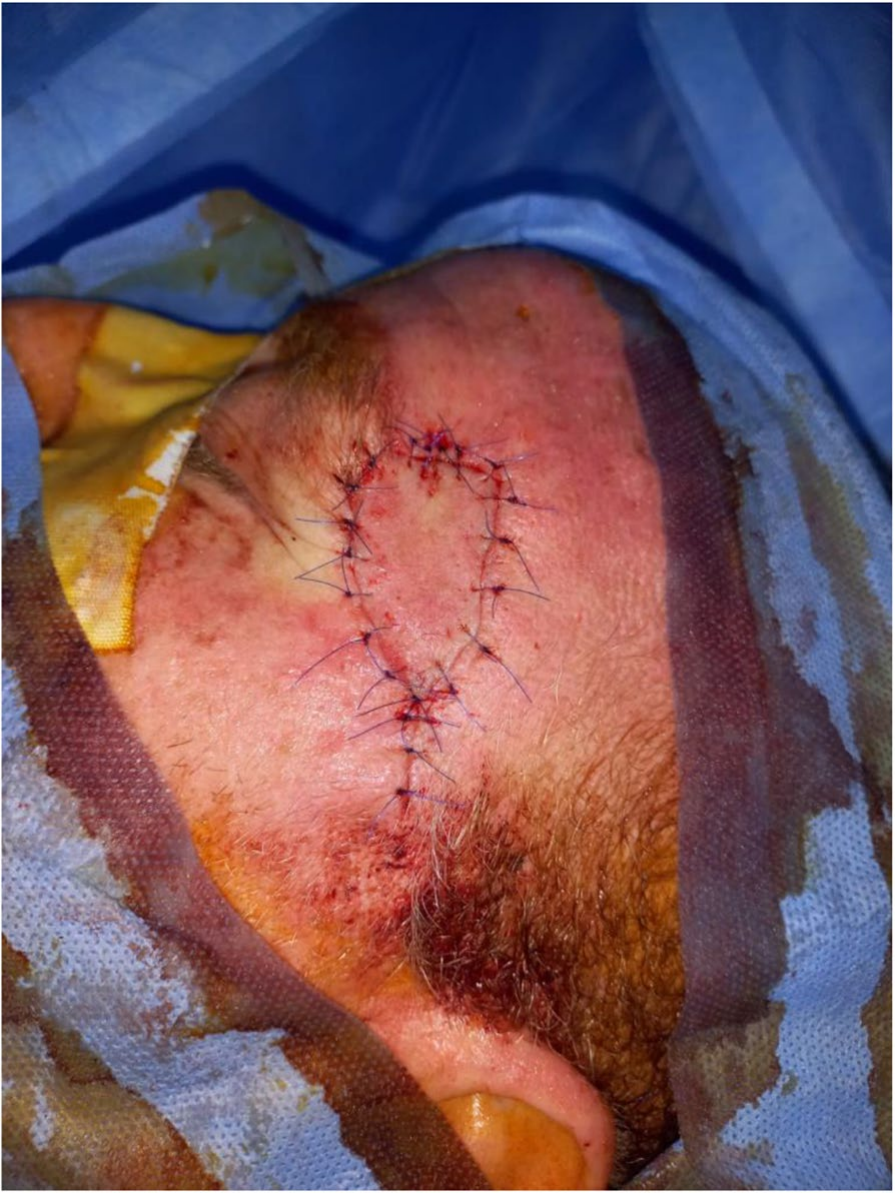
Patient (3): in the setting of the flap

**Fig. 18:**
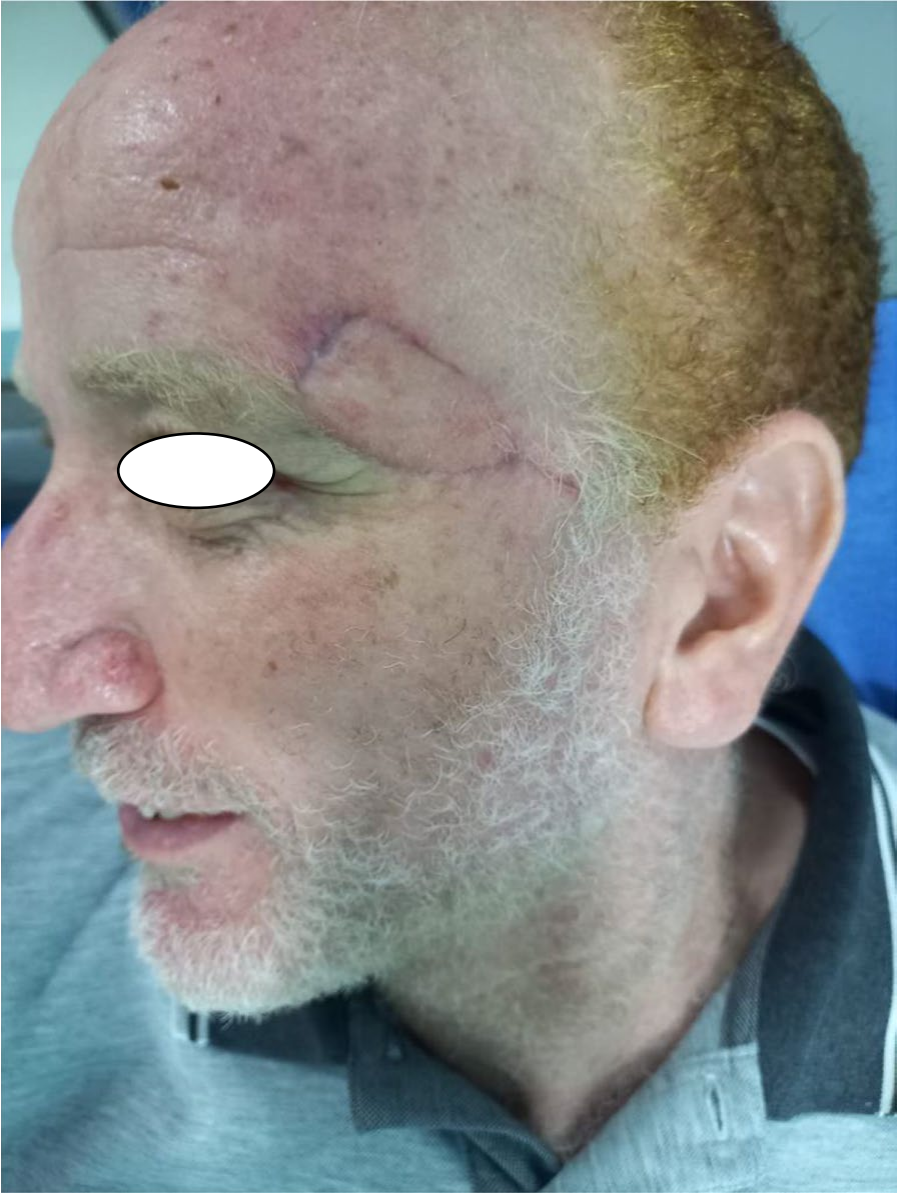
Patient (3): after healing of the flap in post-operative follow-up

Patient 3: a 42-year-old, medically free, ulcer for 2 months (Figs. 15, 16, 17, and 18).

## Data Availability

This is a prospective cohort study including 30 patients presented small to moderate-size facial defects (5–6 cm) caused by post-tumor resection were selected to operate at the Plastic and Reconstructive Surgery Department, Beni-Suef University Hospital, and other private hospitals with mean age 44 years old using perforator-based flaps. The study was done from June 2021 to June 2022 including a 1-year follow-up post-operative. The datasets used and/or analyzed during the current study available from the corresponding author on reasonable request.
